# Fast, inexpensive, and reliable HPLC method to determine monomer fractions in poly(3-hydroxybutyrate-co-3-hydroxyvalerate)

**DOI:** 10.1007/s00253-021-11265-3

**Published:** 2021-05-20

**Authors:** Stefanie Duvigneau, Alexander Kettner, Lisa Carius, Carola Griehl, Rolf Findeisen, Achim Kienle

**Affiliations:** 1 Institute for Automation Engineering, Otto von Guericke University, Magdeburg, Saxony-Anhalt Germany; 2 Department of Applied Biosciences and Process Technology, Anhalt University of Applied Sciences, Köthen, Saxony-Anhalt Germany; 3 Process Synthesis and Process Dynamics, Max Planck Institute for Dynamics of Complex Technical Systems, Magdeburg, Saxony-Anhalt Germany

**Keywords:** Bioplastic, Polyhydroxyalkanoate, Quantitative measurement, High-pressure liquid chromatography, UV/Vis spectrometry

## Abstract

The determination of the monomer fractions in polyhydroxyalkanoates is of great importance for research on microbial-produced plastic material. The development of new process designs, the validation of mathematical models, and intelligent control strategies for production depend enormously on the correctness of the analyzed monomer fractions. Most of the available detection methods focus on the determination of the monomer fractions of the homopolymer poly(3-hydroxybutyrate). Only a few can analyze the monomer content in copolymers such as poly(3-hydroxybutyrate-co-3-hydroxyvalerate), which usually require expensive measuring devices, a high preparation time or the use of environmentally harmful halogenated solvents such as chloroform or dichloromethane. This work presents a fast, simple, and inexpensive method for the analysis of poly(3-hydroxybutyrate-co-3-hydroxyvalerate) with high-performance liquid chromatography. Samples from a bioreactor experiment for the production of poly(3-hydroxybutyrate-co-3-hydroxyvalerate) with *Cupriavidus necator* H16 were examined regarding their monomer content using the new method and gas chromatography analysis, one of the most frequently used methods in literature. The results from our new method were validated using gas chromatography measurements and show excellent agreement.

**Key points**

∙ *The presented HPLC method is an inexpensive, fast and environmentally friendly alternative to existing methods for quantification of monomeric composition of PHBV.*

∙ *Validation with state of the art GC measurement exhibits excellent agreement over a broad range of PHBV monomer fractions.*

## Introduction

Non-degradable waste is polluting our planet more and more. In particular, the accumulation of plastic in marine ecosystems has come into focus (Jambeck et al. 2015). Besides garbage patches on the surface of oceans, there are many smaller plastic fragments in the water. So-called microplastic can be ingested by natural populations and thus, find a way in our food chain. In addition to innovative methods for removing plastic waste from our environment and the tireless educational work of organizations such as the Pacific Garbage Screening (everwave), the durability of conventional plastic is a problem. Therefore, many research groups and companies are concerned with the synthesis of biodegradable plastic raw material (Sabapathy et al. 2020). The biopolymer group of polyhydroxyalkanoates (PHAs) is a promising raw material. These aliphatic polyesters are not only biodegradable but also non-toxic, which allows a wide range of applications (Puppi et al. 2019; Koller and Braunegg 2018; Koller 2014). PHAs can be synthesized by a variety of microorganisms and plants as storage compounds under limited conditions with inexpensive and renewable substrates (Koller et al. 2005, 2010; Brigham and Riedel 2019; Riedel et al. 2014; Koller and Braunegg 2018). One of the best studied PHA is poly(3-hydroxybutyrate) (PHB), a homopolymer of 3-hydroxybutyrate (3HB). In contrast to PHB, the copolymer poly(3-hydroxybutyrate-co-3-hydroxyvalerate) (PHBV) with 3HB and 3-hydroxyvalerate (3HV) as monomers has a lower melting temperature and crystallinity and can be easier processed for industrial applications (Leroy et al. 2012; Aramvash et al. 2016; Zinn et al. 2003).

The correct analysis of the microbially produced PHBV is one basis to increase the amount of PHBV-based applications. In addition to the information about the distribution (Chiellini et al. 2003; žagar and Kržan 2004), the composition of heteropolymers such as PHBV is an important indicator for the thermal and mechanical properties of the raw material. There are a number of analytical methods in literature for determining the monomer content of PHAs (Koller and Rodríguez-Contreras 2015; Tan et al. 2014). Some methods, such as analysis with a transmission electron microscope (TEM) (Thomson et al. 2011) or Nile red staining (Gorenflo et al. 1999; Wu et al. 2003; Pick and Rachutin-Zalogin 2012; Zuriani et al. 2013), focus on the detection of the intracellular granules that contain the PHAs. Beside using harmful chemicals for TEM fixation, absolute quantification after staining with Nile red is often a problem. On the one hand, lipids present in samples falsify the measurement result and a clear correlation between the PHA content and the fluorescence intensity is not always possible. Furthermore, it is not possible to differentiate between different monomers. The same applies to older spectrometric methods without prior chromatographic separation (Law and Slepecky 1960a, 1960b). After extraction and acidic hydrolysis of the cell samples with heteropolymers as storage material, UV spectra of the hydrolysis products of the monomers overlap and therefore, cannot be distinguished. Not only the monomers from hydrolyzed heteropolymers overlap but also other hydrolysis products from the cell have an absorption maximum at 235 nm, which makes error-free analysis even more difficult (Law and Slepecky 1960a).

Gas chromatography (GC) methods provide more detailed and precise measurements (Riis and Mai 1988; Comeau et al. 1988; Braunegg et al. 1978). The accuracy of measurement could be the reason why a lot of research groups use these analysis methods for quantification, although the use of environmentally harmful solvents like chloroform, dichloromethane, or diethylethane is necessary.

A good alternative is provided by liquid chromatography (LC) methods that have already been tested on high-performance liquid chromatography (HPLC) systems (Karr et al. 1983; Hesselmann et al. 1999; Korotkova et al. 1997; Satoh et al. 2016). Various detection systems can be used for analysis, such as UV detectors as commonly used in HPLC systems (Korotkova et al. 1997). In comparison to GC methods, LC methods are less time consuming in view of sample preparation. Further, samples for LC can be performed directly from the culture broth, whereas GC measurements need dried biomass.

**Fig. 1:**
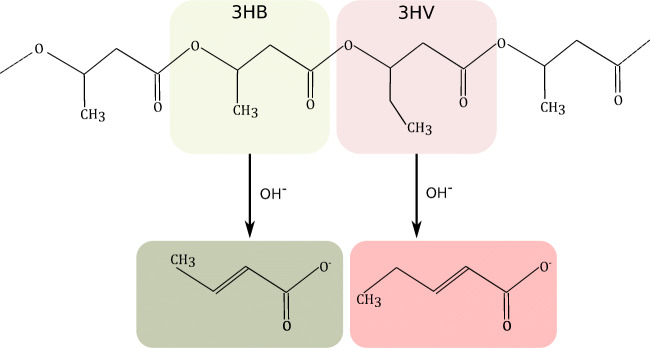
Part of a poly(3-hydroxybuyrate-co-3-hydroxyvalerate) chain and the products after alkaline hydrolysis

In the present work, we demonstrate that neither an expensive ion exchange column (Satoh et al. 2016) nor harmful solvents (Korotkova et al. 1997) are necessary to measure copolymer composition, like 3HB and 3HV contents, in a short and cost-efficient way. For this purpose, culture samples from a bioreactor experiment with *Cupriavidus necator* H16 as well as PHBV test samples with 12% 3HV content are processed. For successful quantification of 3HV with a reverse phase column, an alkaline hydrolysis is required (Satoh et al. 2016; Korotkova et al. 1997; Watanabe et al. 2012). In contrast to acidic hydrolysis, the chemical conversion of 3HV in the polymer takes place due to the higher nucleophilicity of hydroxide ions during alkaline hydrolysis. For this, understanding of the chemical reactions taking place is important (see Fig. 1). As outlined by Watanabe and colleagues (Watanabe et al. 2012), the reaction leading to crotonic or 2-pentenoic acid as product is an E1 elimination/dehydration reaction. A proton is eliminated from the *α*-C atom and a double C-C bond is formed. With acid hydrolysis, only water molecules with low nucleophilicity are available. Furthermore, the electron density at the *α*-C atom increases with increasing length of the side chain. Since an 3HV monomer has an ethyl side chain, the electron density of the *α*-C atom appears to be too high to successfully eliminate the proton by water. However, if hydroxide ions are present, the elimination can take place since those ions have a higher nucleophilicity than water (Watanabe et al. 2012). After successful hydrolysis, the extracts can be separated with a weak acidic eluent on an inexpensive reverse phase column and then quantified regarding their absorption at 210 nm.

All in all, our HPLC method represents an inexpensive, fast, and environmentally friendly alternative to existing methods. Hence, the represented method is a key to increase research on PHBV and consequently to raise the competitiveness of PHBV for industrial applications.

## Methods

### Microorganisms, media, and cultivation

To obtain the microbial PHBV samples, an experiment with *Cupriavidus necator* (H16, DSM 428, DSMZ GmbH Braunschweig) was performed. Bacteria were precultured in a shake flask filled with 10 vol% standard LB medium (Carl Roth, Karlsruhe, Germany). After preculture, the inoculum was transferred in a DASGIP parallel bioreactor system (Eppendorf AG, Juelich, Germany) with 1.2 L working volume, pH-control at 6.8, and dissolved oxygen (DO) control at 70%. The optical density at 600 nm was set to 0.4 at the beginning of cultivation. The temperature in the reactor was kept at 30 °C. Stirrer speed and the gas flow rate were used for the DO control. In the first cultivation phase, a balanced N/C ratio was applied to trigger exponential growth. For this, the M81 medium without vitamin solution (DSMZ, 2011) was supplemented with 20 g/L fructose (Carl Roth, Karlsruhe) and 1.5 g/L ammonium chloride (Carl Roth, Karlsruhe, Germany). Propionic acid (Sigma-Aldrich, St. Louis, USA) had an initial concentration of 0.5 g/L to avoid inhibitory effects (Kim et al. 1992). To reach a high 3HV content in the PHBV copolymer production phase, the pH correction was carried out with 20 g/L propionic acid and 2 N sodium hydroxide (Carl Roth, Karlsruhe, Germany). Samples were collected between 0 and 38 h for analysis. To provide an adequate statistical basis, 6 samples were harvested after 15 h of cultivation, additionally.

### Determination of total biomass

For the determination of total biomass (TBM), 5 mL culture broth was centrifuged 10 min at 3200×g and 4 °C (Allegra X-12R, Beckman Coulter, Brea, USA). In a second step, the cell pellet was lyophilized over night (Alpha 2-4 LDplus, Christ, Osterode, Germany) and weighted. Six replicates of the dried samples were used for the GC-based PHBV monomer determination.

### UV/Vis spectroscopy

The PHA content was measured using the spectrometric method as suggested in Law and Slepecky (1960a). The PHAs of the samples were extracted from the cells with dichloromethane (Carl Roth, Karlsruhe, Germany). Two milliliters of the chlorinated hydrocarbon is added to each previously lyophilized sample and the mixture was boiled in a water bath for 10 min. After cooling down to room temperature, cell pellets were centrifuged for 10 min at 3200×g and 4 °C (Allegra X-12R, Beckman Coulter, Brea, USA) and the supernatants were transferred to a 10-mL glass tube. Afterwards, the extraction step was repeated two times. The supernatants were then evaporated to obtain the extracted PHAs. After complete evaporation of the dichloromethane, samples were boiled with 2 mL of concentrated sulfuric acid (98%, Carl Roth, Kalrsruhe, Germany) in a water bath for 30 min. After cooling down to room temperature, 200 μL of the sample was transferred in a microtiter plate and measured in a spectrometer (Eon, BioTek, Winooksi, USA). Thereby, a spectrum was recorded between 200 and 800 nm using 5 nm slit. The lamp of the device provides monochromatic light in the visual and UV range. The maximum absorbance at 235 nm can be used to determine the amount of crotonic acid in the sample.

### Gas chromatography

The GC-MS analysis of PHBV was performed using a modified procedure based on the findings of Riis and Mai (1988). Five to ten milligrams of dried biomass was simultaneously hydrolyzed and transesterified in sealed headspace vials using 4 mL 1,2-dichloroethane, 2 mL 4:1 (vol/vol) propanol sulfuric acid mixture, and 100 μL of 20 g/L benzoic acid as internal standard. Acidic propanolysis was carried out for 4 h at 120 °C. Then, 4 mL water was added, mixed, and waited until phase reseparation. One millilitre of the lower phase was transferred for GC analysis. Separation took place on a Stabilwax column (Restek, Bad Homburg, Germany) using Helium as carrier at a flow rate of 1.44 mL/min, and a gradient of 120 °C (3 min), 140 °C at 3 °C/min, 230 °C at 50 °C/min, and 240 °C at 10 °C/min. A PHBV standard curve (12% 3HV, Sigma-Aldrich/Merck, Darmstadt, Germany) was used for quantification and qualification of the propionyl-3HB and propionyl-3HV.

### High-pressure liquid chromatography

For the determination of hydroxyalkanoate concentration, 1 mL culture broth was alkaline digested and prepared as reported in Satoh et al. (2016). Further, 10 μL of the digested and filtered samples were loaded on the reverse phase column Inertsil 100A ODS-3 (5 μm pore size, 250 × 4.6mm, MZ-Analysentechnik GmbH, Mainz, Germany) and eluted isocratically with 1 mL/min at 60 °C. The eluent consists of 92% low concentrated sulfuric acid (0.025% solution, Carl Roth, Karlsruhe, Germany) and 8% acetonitrile (Carl Roth, Karlsruhe). The 3HB and 3HV concentrations of the samples were determined by crotonic (Carl Roth, Karlsruhe, Germany) and 2-pentenoic acid standard samples (Sigma-Aldrich, St. Louis, USA), respectively. Since the alkaline digestion does not lead to a complete conversion of 3HB to crotonic acid and 3HV to 2-pentenoic acid, a PHBV sample (12% 3HV, Sigma-Aldrich /Merck, Darmstadt, Germany) with known concentration is needed to calculate the conversion yields *Y*_3*HB*_ and *Y*_3*HV*_ (Satoh et al. 2016):

1 $$  Y_{3HB}=2\cdot\frac{c_{CA}}{c_{3HB}} $$

2 $$  Y_{3HV}=2\cdot\frac{c_{PA}}{c_{3HV}} $$

Here, the dilution ratio (D) is 2, *c*_3*HB*_ is the known 3HB, and *c*_3*HV*_ the known 3HV concentration of the PHBV test sample. The concentrations of crotonic acid *c*_*CA*_ and 2-pentenoic *c*_*PA*_ acid were determined by linear regression of standard concentrations.

The peaks were detected with a UV/Vis diode-array detector (G7115A, Agilent, Waldbronn, Germany) using a slit of 4 nm at 210 nm.

### Statistics

A comparison between the measurement results obtained with GC and HPLC was performed by a paired comparison test using the Origin software (OriginLab Corporation, MA, USA).

## Results

In the present work, an HPLC method is presented which is time and cost efficient, simple, and environmentally friendly. For the chromatographic separation, culture samples were prepared as described in the “Methods” section. In addition to the extraction of the polymer, PHAs are simultaneously hydrolyzed. As already shown in Satoh et al. (2016), the optimal duration and temperature for the hydrolysis is 1 h at 105 °C, which are used here, too. For all steps up to the analysis of the hydrolysis products with HPLC, approx. 1 h and 15 min is required per sample. In comparison to the established GC method (see “Methods” section), the time demand is reduced to approximately 25%. For the presented HPLC method, the active working time for the preparation of one sample is less than 5 min and can be easily integrated into everyday laboratory work.

After centrifugation and filtration, 10 μL of the filtered sample is injected onto a reverse phase column. By using the reverse phase column instead of an ion exchange column as described by Satoh et al. (2016), the costs of this step could be reduced by 85%. Furthermore, reverse phase columns are commonly used in standard HPLC analytic, which makes the integration of the method even easier.

**Fig. 2:**
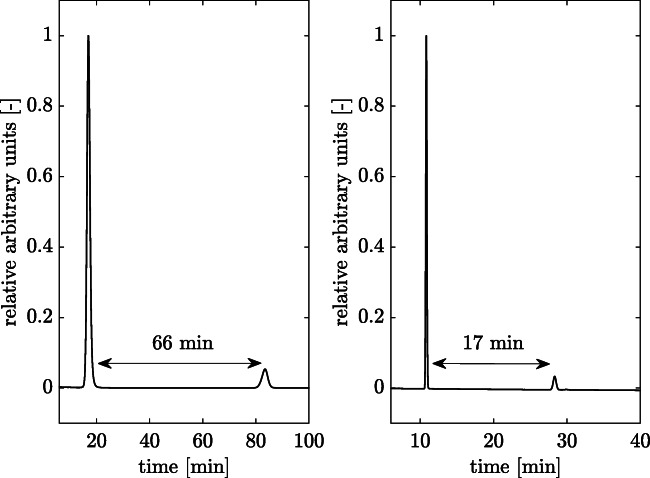
Chromatographic separation with different eluents. HPLC measurements were performed with an isocratic eluent: left panel, 100% H_2_*SO*_4_ (0.0025%); right panel, 92% H_2_*SO*_4_ (0.0025%) and 8% acetonitrile. Ten microliters standard mix consisting crotonic acid (0.05 g/L, early peak) and 2-pentenoic acid (0.01 g/L, later peak) were eluted for both cases

As can be seen in Fig. 2, the run time for the detection of 3HV and 3HB hydrolysis products can be reduced to 26% using the adapted eluent. This can be achieved by adding 8% acetonitrile to 92% low concentrated sulfuric acid (see “Methods” section). The resulting peaks for crotonic acid and 2-pentenoic acid are easily separable (Fig. 2). In addition to dehydration of 3HB and 3HV to crotonic acid and 2-pentenoic acid as product, hydrolysis of the copolymer to the monomers 3HB or 3HV can also take place under alkalic conditions (Yu et al. 2005). Therefore, it is important to calculate conversion yields using the quantified dehydration products and a known polymer concentration and composition (see Eqs. 1 and 2). The PHBV sample can be easily processed together with the samples and standards for determination the conversion yields.

**Fig. 3:**
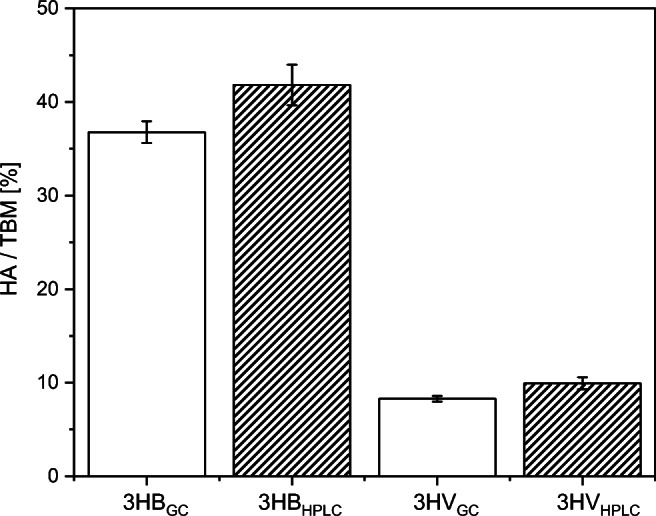
Evaluation of HPLC with a standard GC method. The hydroxyalkanoate monomer (HA) amount of the total biomass (TBM) was measured from six replicates for each measurement method. A paired *t*-test was performed between the two groups. The *p*-values for 3HB and 3HV are 0.11 and 0.16, respectively

Measurements obtained with an established and widely accepted GC method are used for validation of the results obtained with our HPLC method. The comparison for 3HB and 3HV contents is shown in Fig. 3. For this purpose, six samples were taken from the PHBV production phase of a fed-batch cultivation and analyzed using both the HPLC and the GC methods. A *t*-test shows high *p*-values for both 3HB (*p* = 0.11) and 3HV (*p* = 0.16) contents. The null hypothesis “The mean values of the two measurement methods are significantly different” (test *P*-value < 0.05) can only be rejected.

**Fig. 4:**
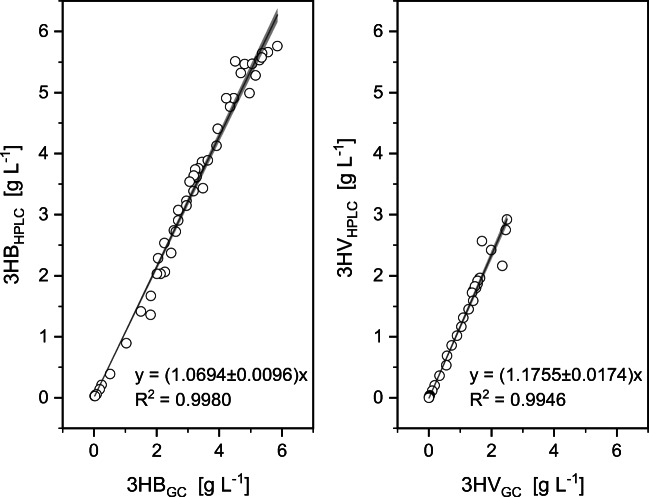
HPLC/GC-correlation of 3HB (left) and 3HV measurements (right). Samples were taken between 0 and 35 h after inoculation and analyzed with HPLC and GC, respectively

In order to check whether the HPLC measurement delivers correct results for low and high PHBV concentrations, samples were taken at regular intervals in the fed-batch experiment with propionate and fructose as carbon sources and analyzed with HPLC and GC. Figure 4 and the corresponding *R*^2^ values show that the measurements using our new method and those from the established GC method exhibit a linear correlation over a wide range. Hence, our method can be used for the quantification of different 3HV/3HB ratios.

**Fig. 5:**
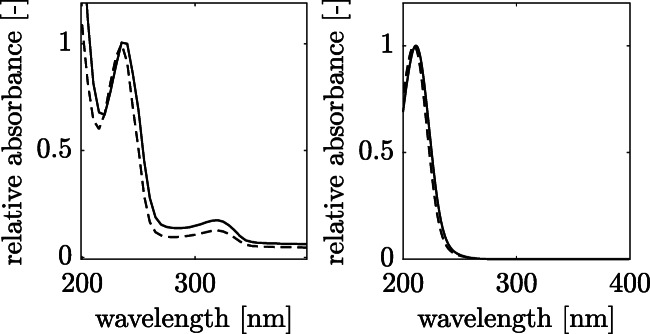
Measurements obtained by UV/Vis spectrometry (left) and HPLC/photodiode-array detector (right). By UV/Vis measurement crotonic (0.01 g/L, dashed lines) or 2-pentenoic acid (0.01 g/L, solid lines) can be detected with an absorbance maximum at 235 nm and 240 nm, respectively. By photodiode-array detection, both substances can be detected with an absorbance maximum at 210 nm

Figure 5 shows a comparison of the spectra obtained with a UV/Vis detector (Fig. 5, left) and with a photodiode array detector (Fig. 5, right). The subordinate spectrum of the concentrated sulfuric acid can be seen in the UV/Vis detection. Furthermore, absorption maxima of the hydrolysis product of 3HB or 3HV can be detected at 235 nm and 240 nm, respectively. Hence, chromatographic separation in the HPLC is necessary for the quantification of PHBV monomers.

## Discussion

Correct analysis of PHAs is essential for the design of competitive microbial polymer production processes. In the current work, the selected representative from the group of microbially produced PHAs is PHBV. The copolymer PHBV has improved processability compared to the homopolymer PHB (Puppi et al. 2019). The proportion of the 3HV monomer influences the thermal and mechanical properties of the product (Shang et al. 2012). To control the properties, the analysis of the polymer composition is essential. The presented HPLC method is cost efficient, quick, and easy to establish. Using our method, monomer proportions of PHBV can be determined very precise over a wide range of different 3HB/3HV ratios. The measurements obtained with HPLC are in very good agreement with the results of a widely established GC measurement.

Future work will focus on the detection of monomers with longer side chains, e.g., medium chain length (*mcl*)-hydroxyalkanoates like 3-hydroxyhexanoate. Further, the applicability of the method for the dectection of 4-hydroxybutyrate should be tested. For polymers consisting monomers with *mcl*, quick and easy detection of those is desirable too and would provide an important step towards successful product design. Furthermore, a long-term test should show whether the conversion yields decrease with the age of the reverse phase column. In addition, the sample volume can be reduced even further by performing the alkaline hydrolysis in microtiter plates as in Watanabe et al. (2012).

Our developed method represents an environmentally friendly, time-, and cost-efficient alternative to existing measurement methods. The resulting precisely determined monomer fractions make it possible to design the underlying fermentation process with regard to desired polymer properties. Hence, our method represents an important step towards the competitiveness of microbial polymers.
